# Immediate and delayed micro shear bond strength evaluation of two glass ionomer cements to composite resin by using different bonding techniques—an in vitro study

**DOI:** 10.1038/s41405-024-00283-8

**Published:** 2024-12-17

**Authors:** Somaya Ali Saleh, Nisreen Nabiel Hassan, Amna Algarni, Ranya Zahran, Abeer Farag, Danya Hashem

**Affiliations:** 1 Department of Operative Dentistry, Faculty of Dentistry, Ain Shams University, Cairo, Egypt; 2 Department of Restorative Dental Science, College of Dentistry, Taibah University, Madinah, Saudi Arabia; 3 Department of Restorative Dentistry, Faculty of Dentistry, Minia University, Minia, Egypt

**Keywords:** Glass-ionomer cement, Composite resin, Bonded restorations

## Abstract

**Objective:**

Evaluating immediate and delayed micro shear bond strength (µSBS) between composite resin and glass ionomer cements using different adhesive systems and mechanical surface treatment.

**Materials and methods:**

A total of 240 specimens of glass ionomer restorative materials were divided into two groups: Resin Modified Glass Ionomer Cement (RMGIC) namely Riva Light Cure and Conventional Glass Ionomer Cement (CGIC) namely Riva Self Cure. These were subdivided into immediate (24 h) and delayed (3 months) storage and further divided into smooth, medium, and rough surface treatment with either total etch (TE) or self-etch (SE) adhesive strategies. Composite resin was applied and µSBS of the sample was determined and failure modes were examined.

**Results:**

Immediate µSBS of RMGIC was superior than CGIC and TE was better than SE. Within RMGIC, smooth surface has significantly higher bond strength than medium and rough stone surface treatment. Delayed µSBS of RMGIC was superior than CGIC. Within RMGIC specimens, TE and smooth and medium grit had significantly better bond strength than SE and rough grit. Within CGIC, statistically higher bond strength values were found with medium grit compared to smooth while no difference was found between TE and SE.

**Conclusion:**

Bonding composite resin to smooth RMGIC using TE yielded higher bond strength values than CGIC regardless of the time. Bonding composite resin immediately to CGIC is best done using a TE technique. However, delayed bonding to CGIC requires roughening of the CGIC surface prior to placement of the composite resin to obtain improved bonding.

## Introduction

Dental restorative materials are intended to replace lost tooth structure with materials that are compatible with the oral environment and have enough strength to endure the stress produced during mastication. In 1972 Wilson and Kent have introduced Glass-Ionomer Cements (GICs) to dentistry and since then, they are commonly used in modern dentistry and are well recognized for their advantages, physical and chemical properties [1, 2]. Among these properties, their biocompatibility with the pulp, anticariogenic activity, low shrinkage, coefficient of thermal expansion and fluoride release are considered to be the most important advantages of GICs [3]. Nevertheless, GICs are frequently used as a liner beneath resin composite restorations to seal the dentin and its dentinal tubules and reduce microleakage at the restoration margin. This has been reported to increase the clinical success of the restoration [1, 4]. However, lack of chemical bonding between composite resin and conventional GICs affects the longevity of the final restoration [5].

Accordingly, to improve the clinical application, bonding and mechanical properties of conventional GICs, hydrophilic monomers and the functional group HEMA [hydroxyl-ethyl methacrylate] have been added to GIC forming resin-modified glass ionomer cement (RMGIC) [6, 7]. It was shown that RMGICs have much higher flexural strength and improved bonding to composite resin compared to conventional GICs [8].

The bond between conventional GICs and composite resin is micromechanical. One method to optimize this bond is to create porosities on the surface of the GICs during the bonding process by total acid etching using phosphoric acid (etch-and-rinse systems) which improves the micro mechanical retention [9]. Etching time is another factor that affects the quality of the bond between composite resin and conventional GICs. Currently, sandwich technique restorations are a two-stage procedure that should be completed within 3–6 months in case of deep caries management re-entry techniques. The sandwich technique offers multiple clinical solutions for deep cavities, hyperemic tooth and reversible compromised pulp, and could be used either immediate within the first 24 h or delayed after 2 weeks at least [10–12].

Nowadays, there is a high demand on using self-etch bonding systems which contain acidic monomers. These systems eliminate the step of etch and rinse because they do not necessitate washing step, resulting in improved clinical efficacy [13]. In addition, self-etch adhesive systems contain one or more carboxylic or phosphate groups, which have been shown in studies to have enamel and dentin bond strength similar to that of total acid-etching (etch-and-rinse) adhesive systems [14, 15]. Furthermore, to increase the bonding reliability of GIC to composite, various mechanical conditioning procedures are used; air abrasion using Al_2_O_3_, photodynamic therapy and laser, abrasive stones which boosts surface energy, roughness, and bonding area, hence increasing bond strength [16].

Correlating laboratory tests to clinical performance is challenging as there are no accurate tests for long-term clinical performance of restorative materials. Micro-tensile bond strength (μTBS) is a reliable method for measuring bond strength, as it is less likely to include interfacial flaws due to the small surface area employed. However, this test is technically hard and not suitable for assessing brittle materials due to the need to section specimens into sticks or hour-glass shapes. However micro-shear bond strength (μSBS) testing allows simpler specimen preparation with a reduced risk of specimen preparation damage [12, 17].

Many studies have investigated the bond strength of GIC and RMGIC to composite resin when used in a sandwich technique. However, new improved materials are continuously introduced to the market. Most recently, a new group of glass ionomer and light cured RMGICs have been introduced, all with varying recommendations and abilities. Furthermore, many surface treatment strategies have been suggested using different adhesive methods. This study aims to identify the best surface treatment protocol and adhesive strategy between more recently introduced glass ionomer cements and composite resin when used in a sandwich technique by and resin modified) using; total etch (TE) and self-etch (SE) adhesive systems and employing different mechanical surface treatment. The null hypothesis states that there is no significant different in µSBS between immediate and delayed glass ionomer cements and resin composite restoration utilizing different adhesion strategies and different mechanical surface-treatment.

## Materials and methods

### Study design

This study followed a factorial 2 × 2 × 3 repeated measures design to assess µSBS between composite resin restoration and GIC-based restorative materials. The experimental factors included: (1) type of GI restorative material in 2 levels (RMGIC and CGIC); (2) adhesion strategy in 2 levels (TE and SE); (3) surface condition in 3 levels (smooth, medium and rough). Micro shear bond strength was evaluated both immediately and delayed after aging.

### Sample size

Based on the results of a prior study [18], a power analysis was performed with an alpha (α) and (β) level of (0.05) (i.e., power = 95%) and an effect size (f) of (1.22) determined; where the smallest required sample size (n) was found to be (36) samples. G*Power version 3.1.9.7 was used to calculate sample size.

### Specimen preparation

The materials used are summarized in Table 1. A total of 240 specimens of glass ionomer cement were divided into two groups: *n* = 120 Resin Modified Glass Ionomer Cement (RMGIC) namely Riva light cure® (SDI, Victoria, Australia, *n* = 120 Conventional Glass Ionomer Cement (CGIC) namely Riva self- cure (SDI, Victoria, Australia). They were prepared by mixing each material according to manufacturer’s instructions and condensed into rubber molds with 10 × 10 mm and 4 mm depth, supported over a glass slab base to avoid any error. Celluloid strips were used to cover the materials, and small glass slides were placed above the molds to ensure all the materials were laid against a smooth surface to achieve standardization of the sample surface [18]. Riva light cure® specimens were photo polymerized with an LED polymerization equipment (Ivoclar Vivadent Inc., Amherst, N.Y., USA) at 800 mW/cm2 for 20 s. Riva self-cure specimens were allowed to set for 6 min (regular setting time). All specimens were kept in labeled bottles of distilled water at 37 °C, the bottles were placed in an incubator (Jiangsu XCH Biomedical Technology Co., Ltd., Taizhou, China) and subdivided according to the aging time into immediate (24 h storage) and delayed (3 months storage). All the steps were done by the same operator.

**Table 1:** List of materials used in the study.

Product	Composition	Manufacturer	Lot number
Resin modified glass ionomer cement. Riva light cure	Fluoroaluminosilicate glass powder Polyacrylic acids, Tartaric acid 2-Hydroxyethyle methacrylate Dimethacrylate cross- linker Acidic monomer	SDI, Victoria, Australia	J2102227
Conventional glass ionomer cement. Riva self-cure	Fluoroaluminosilicate glass powder Polyacrylic acids Tartaric acid	SDI,Victoria, Australia	B2208044EA
Composite resin Tetric N ceram	dimethacrylates (19–20 wt%). The fillers contain barium glass, ytterbium trifluoride, mixed oxide and copolymers (80–81 wt%).	Ivoclar Vivadent Inc., Amherst, N.Y., USA	Z01WT9
Universal Adhesive Tetric N -bond universal	Phosphoric acid acrylate, HEMA, Bis-GMA, urethane dimethacrylate, ethanol, film-forming agent, initiators and stabilizers.	Ivoclar Vivadent Inc., Amherst, N.Y., USA	Z030W1
N-etchant	Phosphoric acid (37 wt% in water), thickeners and pigments.	Ivoclar Vivadent Inc., Amherst, N.Y., USA	Z01xth

### Micro shear bond strength test

**Fig. 1:**
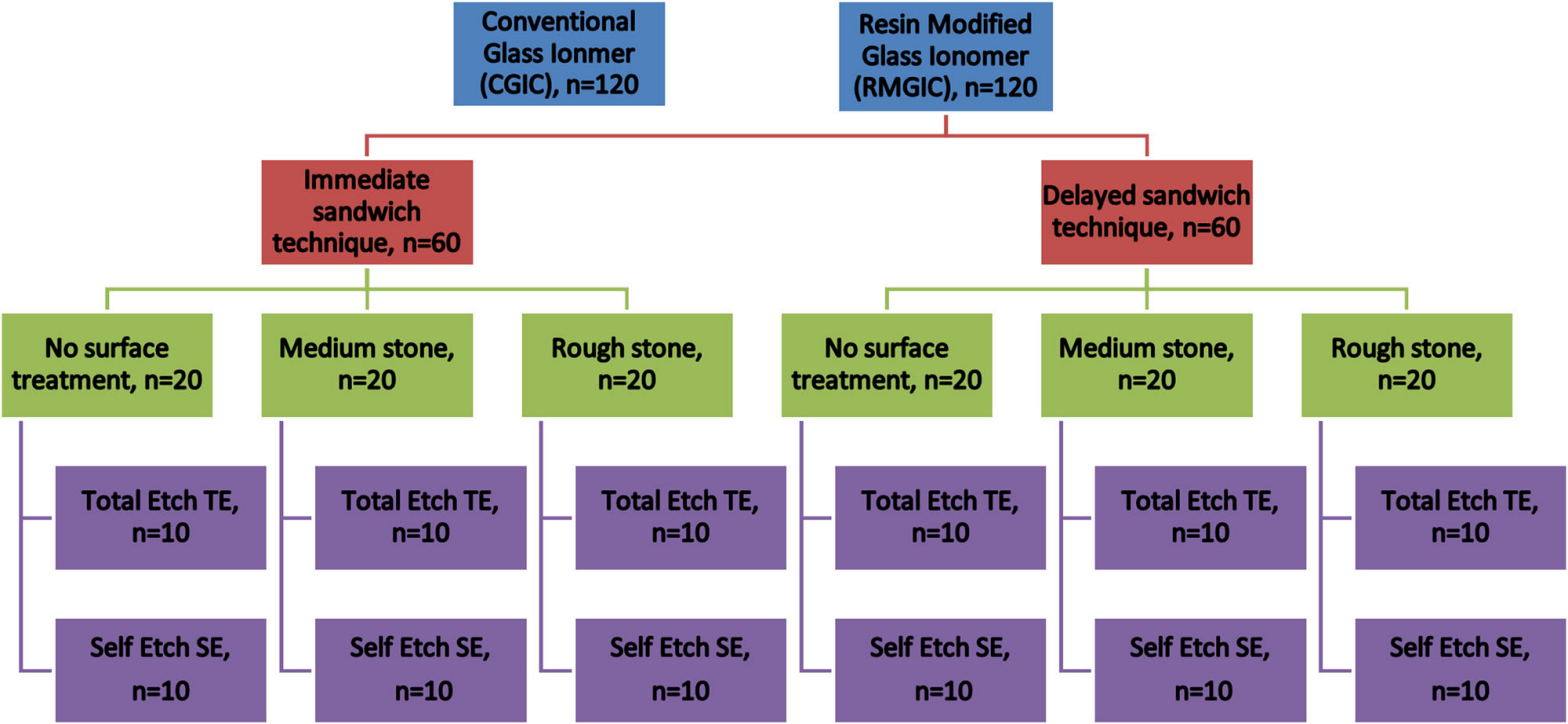
Flow chart illustrating the distribution of specimens into groups and subgroups.

For the immediate tested specimens (*n* = 120), each group was divided into three subgroups according to surface treatment of glass ionomer specimens as follows: group 1: no surface treatment (smooth) (*n* = 40), group 2: medium abrasive stone with particle size 107–126 μm (*n* = 40), group 3: coarse abrasive stone with particle size 151 μm (*n* = 40), (Komet Dental. Gebr. Brasseler GmbH & Co.KG. Germany). Each subgroup was further divided into two groups consisting of 20 specimens each according to different etching strategies but using one universal adhesive (Tetric N -bond universal ®Ivoclar Vivadent, Inc., Amherst, N.Y., USA). Group (a): self -etch (*n* = 20) using only the universal adhesive (Tetric N -bond universal ®). (b): total etch (*n* = 20), using 37% phosphoric acid N-etchant gel (Ivoclar vivadent, Inc., Amherst, N.Y., USA) then the universal adhesive (Fig. 1).

A hollow translucent polyethylene tube of 0.8 mm in diameter and 2 mm height was placed over each specimen after adhesive application (agitation for 20 s, solvent evaporation for 5 s and light curing for 10 s) and filled with composite resin (Tetric N -ceram®, Ivoclar Vivadent Inc., Amherst, N.Y., USA) and light cured for 10 s Specimens were then subjected to micro shear bond test using Hounsfield Universal testing machine (Instron, USA) at a cross head speed of 1 mm/min.

For the delayed tested specimens (*n* = 120), the same groups and preparation methods were applied after 3 months storage of the RMGIC and CGIC specimens in distilled water.

### Stereomicroscope examination

To identify the failure mode, all de-bonded surface samples were inspected using a 40-magnification stereomicroscope (Olympus SZX16, Olympus, Tokyo, Japan). These were divided into three categories: mixed, adhesive, and cohesive failure. If the adhesive interface and the restorative substance (GIC substrate or resin composite material) were incorporated, mixed failure was observed. Adhesive failure was detected if it occurred at the GIC/adhesive interface, even if minute amounts of adhesive resin were visible on the GIC substrate. Cohesive failure was considered if it occurred inside the GIC substrate or the resin composite.

### Statistical analysis

Means with 95% confidence intervals, standard deviation (SD), minimum and maximum values were used to display numerical data. The Shapiro–Wilk test was employed to determine normality. Levene’s test was used to determine variance homogeneity. The data had a parametric distribution and homogeneous variance and were analyzed using three-way ANOVA followed by Tukey’s post hoc test. The error term of the three-way model with *p*-values adjusted using Bonferroni correction was used to compare simple main effects. Within all tests, the significance level was set at *p* < 0.05. Statistical analysis was performed using “R” statistical analysis software version 4.3.0 for Windows. R core team (2024) R: A language and environment for statistical computing. R Foundation for statistical computing, Vienna, Austria. URL https://www.R-project.org/.

## Results

### Immediate µSBS evaluation

**Fig. 2:**
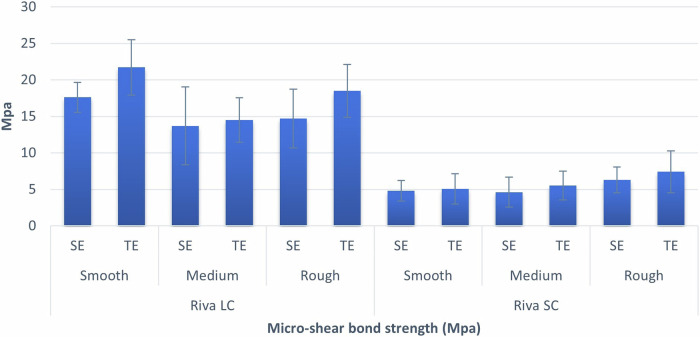
Bar chart demonstrating immediate mean and standard deviation values (error bars) of µSBS for different variables.

Table 2 and Fig. 2 show descriptive statistics for immediate µSBS values. Table 3 displays the results of three-way ANOVA. The findings revealed that the type of adhesive strategy has a statistically significant effect on bond strength with samples treated with TE system having significantly higher values (*p* = 0.001) compared to SE. In addition, there was a statistically significant interaction between material type and finishing stone (*p* < 0.001). Simple effects comparison for material and finishing stone grit were carried out. Results showed that regardless of the type of stone used, significantly higher bond strength values were achieved with Riva LC (*p* < 0.001). They also showed that for Riva LC samples, there was a significant increase in bond strength with the decrease of the grit roughness (*p* < 0.001), while for Riva SC samples, stone grit had no effect on bond strength (*p* = 0.089).

**Table 2:** Immediate descriptive statistics for µSBS (MPa).

Material	Finishing stone grit	Conditioning	Mean	95% CI^a^		SD^b^	Min.	Max.
				Lower	Upper			
Riva LC	smooth	SE	17.60	16.32	18.88	2.07	15.00	21.00
		TE	21.70	19.36	24.04	3.77	17.00	28.00
	Medium	SE	13.70	10.41	16.99	5.31	8.00	21.00
		TE	14.50	12.60	16.40	3.06	10.00	18.00
	Rough	SE	14.70	12.22	17.18	4.00	10.00	21.00
		TE	18.50	16.25	20.75	3.63	12.00	25.00
Riva SC	smooth	SE	4.80	3.93	5.67	1.40	2.00	7.00
		TE	5.06	3.79	6.34	2.06	2.00	8.62
	Medium	SE	4.62	3.35	5.90	2.06	2.00	8.25
		TE	5.51	4.29	6.74	1.98	2.00	8.12
	Rough	SE	6.30	5.20	7.40	1.77	3.00	9.00
		TE	7.40	5.62	9.18	2.88	3.00	11.00

*SD* standard deviation.

^a^95%CI = 95% confidence interval for the mean.

**Table 3:** Three-way ANOVA for immediate µSBS values.

Parameter	Sum of squares	df	Mean square	*f*-value	*p*-value	Partial eta squared (95% CI)
Material	3740.83	1	3740.83	404.33	<0.001*	0.789 (0.731:0.826)
Finishing stone grit	163.01	2	81.51	8.81	<0.001*	0.140 (0.046:0.232)
Conditioning	99.92	1	99.92	10.80	0.001*	0.091 (0.023:0.183)
Material* stone grit	191.84	2	95.92	10.37	<0.001*	0.161 (0.061:0.256)
Material* conditioning	34.67	1	34.67	3.75	0.056	0.034 (0.000:0.105)
Stone grit * conditioning	14.80	2	7.40	0.80	0.452	0.015 (0.000:0.059)
Material* stone grit* conditioning	20.39	2	10.20	1.10	0.336	0.020 (0.000:0.070)
Error	999.20	108	9.25			

*significant (*p* < 0.05), eta < 0.02 - Very small, 0.02 <= eta < 0.13 – Small, 0.13 <= eta < 0.26 – Medium, eta >= 0.26 - Large.

**Fig. 3:**
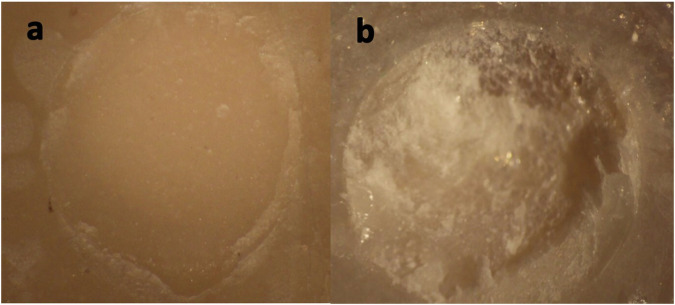
Stereomicroscope images of the samples illustrating failure mode. **a** Cohesive failure (**b**) Mixed failure.

The analysis of fracture modes across the immediately tested specimens revealed the prevalence of cohesive failure for both types of GICs. Riva LC specimens displayed 90% (*n* = 54) cohesive failure (Fig. 3a) and Riva SC groups displayed 91.66% (*n* = 55) cohesive failure applied in either SE or TE bonding modes and regardless the stone grits. The percentages of cohesive failure according to the bonding techniques are: (46%) Riva LC SE, (44%) Riva LC TE, (43%) Riva SC SE, and (47%) Riva SC TE.

Mixed failure mode was observed for both types of GICs when applied after no surface treatment and with medium stone grit. A total of 10% (*n* = 6) showed mixed failure between universal adhesive and Riva LC, while 8.33% (*n* = 5) showed mixed failure between universal adhesive and Riva SC (Fig. 3b).

### Delayed µSBS evaluation

**Fig. 4:**
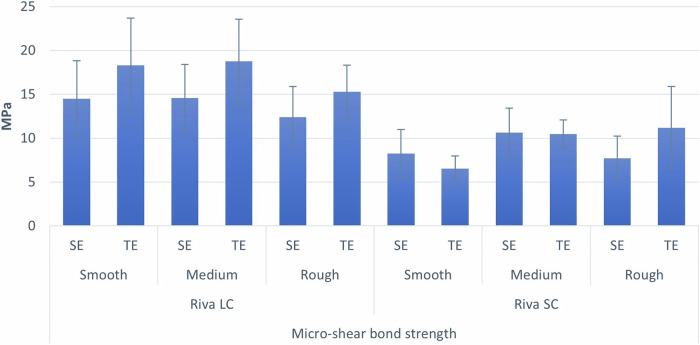
Bar chart demonstrating delayed mean and standard deviation values (error bars) of µSBS for different variables.

Table 4 and Fig. 4 show descriptive statistics for delayed µSBS values. Table 5 displays the results of three-way ANOVA. The findings revealed that there were significant interactions between material types-stone grit (*p* = 0.018) and between material type-conditioning system (*p* = 0.020). Simple effects comparison for material and finishing stone grit was carried out. Results showed that regardless of the type of stone used, significantly higher µSBS values were achieved with Riva LC (*p* < 0.001). Furthermore, Riva LC samples finished with rough stone were found to have significantly lower µSBS values than those finished with smooth and medium stones (*p* < 0.001). Finally, Riva SC samples finished with medium grit stone were found to have significantly higher µSBS values than those finished with smooth stone (*p* < 0.001). Simple effects comparison for material and conditioning system were carried out. Results showed that regardless of conditioning type, significantly higher µSBS values were achieved with Riva LC (*p* < 0.001). They also showed that Riva LC samples conditioned with a TE system had significantly higher µSBS values compared to SE samples (*p* < 0.001). While in the case of Riva SC samples, the conditioning system had no significant effect on the µSBS (*p* = 0.574).

**Table 4:** Delayed descriptive statistics for µSBS (MPa).

Material	Finishing stone grit	Conditioning	Mean	95% CI		SD	Min.	Max.
				Lower	Upper			
Riva LC	Smooth	SE	14.48	11.78	17.18	4.36	8.70	21.79
		TE	18.31	14.96	21.66	5.40	11.50	27.73
	Medium	SE	14.59	12.23	16.96	3.81	9.40	20.55
		TE	18.78	15.81	21.75	4.79	8.58	25.00
	Rough	SE	12.40	10.23	14.56	3.50	8.18	17.56
		TE	15.28	13.40	17.17	3.04	9.80	18.70
Riva SC	Smooth	SE	8.26	6.55	9.96	2.75	3.00	11.30
		TE	6.54	5.65	7.43	1.44	4.30	8.25
	Medium	SE	10.66	8.94	12.38	2.77	6.00	14.20
		TE	10.47	9.47	11.47	1.61	7.22	12.77
	Rough	SE	7.72	6.14	9.29	2.54	4.70	12.44
		TE	11.20	8.29	14.10	4.68	2.10	18.84

*SD* standard deviation

95%CI = 95% confidence interval for the mean

**Table 5:** Three-way ANOVA for delayed µSBS values.

Parameter	Sum of squares	df	Mean square	*f*-value	*p*-value	Partial eta squared (95% CI)
Material	1267.90	1	1267.90	97.91	<0.001*	0.475 (0.362:0.561)
Finishing stone grit	92.78	2	46.39	3.58	0.031*	0.062 (0.003:0.137)
Conditioning	129.66	1	129.66	10.01	0.002*	0.085 (0.019:0.176)
Material* stone grit	108.67	2	54.33	4.20	0.018*	0.072 (0.007:0.150)
Material* conditioning	72.52	1	72.52	5.60	0.020*	0.049 (0.004:0.128)
Stone grit * conditioning	22.72	2	11.36	0.88	0.419	0.016 (0.000:0.062)
Material* stone grit* conditioning	53.16	2	26.58	2.05	0.133	0.037 (0.000:0.099)
Error	1398.61	108	12.95			

*significant (*p* < 0.05), eta < 0.02 - Very small, 0.02 <= eta < 0.13 – Small, 0.13 <= eta < 0.26 – Medium, eta >= 0.26 – Large.

The analysis of fracture modes across delayed tested specimens revealed cohesive failure modes in both types of GICs, with a higher occurrence in the SE bonding mode (95%) compared to the TE bonding mode (85%), regardless of the stone grit used. Specifically, the Riva LC group exhibited a cohesive failure rate of 93.33% (*n* = 56), while the Riva SC group demonstrated a cohesive failure rate of 96.66% (*n* = 58).

In addition, mixed failure modes were observed for both types of GICs when applied after medium stone grit surface treatment compared to other surface treatments. Notably, 6.66% (*n* = 4) of the specimens exhibited mixed failure between the universal adhesive and Riva LC, whereas 3.33% (*n* = 2) exhibited mixed failure between the universal adhesive and Riva SC.

## Discussion

The sandwich technique is commonly used in restoring teeth in operative dentistry to prevent pulp insults and reinforce the remaining tooth structure against masticatory forces. The concept behind this procedure is to combine two restorative materials to create a single strong and durable restoration. As it combines the dentin adhesion and biocompatibility of glass ionomer, as well as the esthetics and mechanical strength of composite resin [19, 20], this has the advantage of acquiring the beneficial physical and esthetic features of each material.

Micro Shear Bond Strength (µSBS) testing is the most preferred and simple way of determining bond strength. This is typically performed to evaluate the bonding strength of dental materials to dentin and between dental materials themselves [21, 22]. This study evaluated immediate and delayed µSBS between two different glass ionomer cements and composite resin after different mechanical surface treatments and adhesive strategies.

The null hypothesis was rejected because there was a significant difference in immediate and delayed µSBS between composite resin and glass ionomer cements using different adhesion strategies and mechanical surface-treatment.

The highest µSBS were found between RMGIC and resin composite under all tested conditions. This finding is consistent with previous reports where RMGIC exhibited higher bond strength to resin composite compared to conventional GIC. This could be due to lack of chemical bonding between CGIC and resin composite materials, as well as water sensitivity of CGICs especially when an immediate sandwich technique is performed. Incorporating a phosphate-based monomer into the liquid phase improved mechanical and adhesive properties of RMGIC [23, 24]. The resin components of RMGIC (e.g., Hydroxyethyl methacrylate (HEMA) could be responsible for the increase in µSBS with resin composite. HEMA is a low-molecular-weight hydrophilic monomer that is readily soluble in water, acetone, and ethanol. It is frequently used in adhesive formulations. Using a resin-based bonding agent, the HEMA component in RMGIC produces better chemical adhesion to the composite system [25]. HEMA molecules incorporated in RMGIC and the oxygen inhibition layer on the surface of RMGICs with the unreacted methacrylate groups, together could create strong chemical covalent bonds with the adhesive resin enhancing the µSBS of the two materials together [26, 27]. Reduced µSBS values of conventional GIC was reported in this study when tested immediately compared to delayed which could be attributed to its sensitivity to moisture immediately after placement [21].

The treatment of the CGIC surface with phosphoric acid in TE adhesion strategy may have resulted in hydration and dehydration of the material leading to subsequent micro cracks and bond failure with resin composite [23]. According to some studies, SBS of RMGIC can be improved further by phosphoric acid surface treatment, air abrasion by Al_2_O_3_ particles and laser application [16, 21] TE approach provided higher µSBS values for RMGIC compared to SE, regardless of any other variable in both immediate and delayed sandwich technique. Acid etching enhances surface wettability of the resin composite and increase the surface energy of the RMGIC due to resin tags infiltration [18]. On the other hand, with CGIC, no difference between TE and SE bonding techniques was found on the reliability of µSBS to resin composite. This could be explained by the use of the same adhesive in both SE and TE modes consistently which excluded the effect of adding another material variable. The nearly similar µSBS values between SE and TE adhesive modes may be attributed to the porous nature of the CGIC surface which may have nullified the effect of the differences between SE and TE bonding techniques [12]. A prior investigation on the effect of etching on CGIC and RMGIC found structural and chemical alterations on the etched surfaces versus non-etched surfaces, however, this had no effect on the micro-hardness of the material. Etching caused surface modifications on the CGIC and to a lesser extent on the RMGIC but with no physical or chemical changes to both materials [28]. On the contrary, several studies reported that mild- etching (a pH of approximately 2.5–3.0) using SE adhesive systems improved bond strength between GIC and composite when compared with TE adhesive systems [29–31]. This was due to strong acids which caused higher neutralization and development of weak fragile salts on CGICs which have a negative impact on bond strength.

Furthermore, the results of the current study showed that within RMGIC, smooth (no treatment) produced significantly higher bond strength values than medium stone surface treatment which in turn is higher than rough stone surface treatment in both immediate and delayed sandwich techniques which is consistent with previous studies [32]. This could be explained by RMGICs’ surface integrity which may be affected by the polishing process creating a weak zone with cracks affecting the glass particles created on the surface of the set RMGIC and abrasion of the matrix [33]. Furthermore, polishing results in the complete or partial removal of the methacrylate groups in the oxygen inhibited layer resulting in lower bond strength values whereas RMGIC left with no polishing has a resin-rich layer on its surface contributing to superior bond strength [32].

In this study, cohesive failure in both RMGIC and CGIC was relatively common rather than adhesive or mixed failure. When this type of failure happens, the true strength of the interfacial bond between the GIC and the resin composite is not evaluated., but actually is a reflection of the cohesive strength of the GICs which is considered a limiting factor in bond strength tests [27]. Nevertheless, this indicates that the bond strength between the composite resin and the GIC was greater than the GIC’s cohesive strength [31]. When the TE adhesive system was employed on the CGIC, surface charged particles may be dissolved by phosphoric acid etching, causing a zone of vulnerability that resulted in material cohesive failure and perhaps decreased bond strength [34]. This may explain the more cohesive failure modes in CGIC observed in the present study. Similarly, RMGIC also exhibited significantly higher cohesive failure modes which may be explained by the superior bond between RMGIC and resin composite interface due to the similarity of resinous monomers in their formulations.

Immediate and delayed µSBS of RMGIC was superior than CGIC with no significance difference in both time intervals which is consistent with other studies [35, 36]. This may be explained by the presence of resin content and early setting reaction of RMGIC due to dual setting reaction with less initial porosity and possible water uptake. Meanwhile, CGICs can be more sensitive to moisture, which may affect their bond strength although maturation with time increases the surface hardness due to the ionic cross-linking and the formation of insoluble polysalt matrix over time [35]. The superior bond strength of RMGICs can contribute to their longevity and resistance to degradation over time, which is crucial for clinical outcomes. While both materials may show similar performance in terms of bond strength at specific intervals, RMGICs may retain their strength better under clinical conditions [35].

This is an in-vitro study; therefore, clinical evaluation and patient follow-up are required to assess the durability of composite resin and GIC bonding when using different adhesive systems with hard tooth structure. The intraoral environment is dynamic, with dental materials subjected to pH fluctuations, salivary enzymes, water sorption, and other variables that may modify the material’s composition and hence influence bond strength. Other protocols may be used in future in-vitro studies that simulate the oral environment such as thermocycling.

## Conclusion

Considering the limitation of this study, bonding of composite resin to RMGIC using TE technique on untreated surface yielded higher µSBS values compared to CGIC, regardless of the time. Immediate bonding of composite resin to CGIC yielded higher µSBS values when using TE with no difference in surface treatment. However, delayed bonding to CGIC requires roughening of the CGIC surface prior to placement of the composite resin to obtain improved bonding regardless of the etching technique used.

## Data Availability

All data included in this study are available from the corresponding author upon request.
